# The influence of reported ADHD and substance abuse on suicidal ideation in a non-clinical sample of young men

**DOI:** 10.1007/s40211-016-0198-6

**Published:** 2016-10-06

**Authors:** Julia Huemer, Anita Riegler, Sabine Völkl-Kernstock, Alexander Wascher, Otto M. Lesch, Henriette Walter, Katrin Skala

**Affiliations:** 1 Dept. of Child and Adolescent Psychiatry, University of Vienna, Waehringer Guertel 18–20, 1090 Vienna, Austria; 2 Dept. of Psychiatry, University of Vienna, Vienna, Austria; 3 Yeshiva University, New York, USA

**Keywords:** ADHD, Suicidal Ideation, Substance Abuse, Adolescents, ADHD, Suizidgedanken, Substanzmissbrauch, Jugendliche

## Abstract

This study intended to determine whether former and current ADHD symptomatology is associated with suicidal ideation in a non-clinical sample of 18 year old males. We performed a cross sectional descriptive study of 3280 men during the examination for military service. The investigation included a screening for substance abuse, past (WURS) and current (ADHD symptom checklist) ADHD symptomatology and an interview about suicidal ideations. We found a correlation of suicidal ideations with a history of ADHD symptomatology. ADHD symptoms were strongly consistent over time. These results indicate that a history of (diagnosed or undiagnosed) ADHD could be a predictor for suicidal ideations. Surveying a history of ADHD in primary care might help identify subjects at risk for suicidal tendencies.

## Zusammenfassung

Im Rahmen dieser Untersuchung sollten in einer Gruppe 18-jähriger Männer untersucht werden, ob und inwiefern anamnestische und gegenwärtige ADHD Symptomatik mit Suizidgedanken assoziiert sind. In einem deskriptiven Querschnittsdesign wurden bei 3280 Männern während ihrer Stellungsuntersuchung ein Screening für Substanzmissbrauch und ein Interview bezüglich Suizidgedanken durchgeführt sowie anamnestische (WURS) und gegenwärtige (ADHD symptom checklist) erhoben. Es zeigte sich ein Zusammenhang zwischen Suizidgedanken und dem Vorhandensein von ADHD-Symptomatik, weiters eine zeitliche Stabilität von ADHD-Symptomatik. Diese Ergebnisse deuten darauf hin, dass die Präsenz von (diagnostiziertem oder undiagnostiziertem) ADHD ein Prädiktor für Suizidgedanken sein könnte. Die Erhebung einer ADHD-Anamnese könnte bei der Identifizierung von Personen mit Suizidimpulsen hilfreich sein.

## Introduction

Attention Deficit-Hyperactivity Disorder (ADHD) is a chronic developmental psychiatric disorder affecting 3–12 % of all children and around 1–6 % of adults [7, 15, 49]. With core symptoms of inattention, impulsivity and hyperactivity the condition often leads to severe social and academic problems [12, 42] thus seriously impairing the lives of those concerned. A growing body of evidence suggests that ADHD is a risk factor for premature death caused by accidents, violence, substance misuse and poor health habits [6, 14, 23]. While factors like a personal or family history of mental illness or suicide, a personal history of sexual, physical or verbal abuse or a history of recent loss are still considered the most important risk factors for suicidal behavior in youth [45], ADHD has been shown to play an important role in the risk of self-harm or suicide [4, 22, 38, 47]. An increased prevalence of ideas of death and suicidal ideation [4] as well as attempted and completed suicide [38] in subjects affected by ADHD has been reported by several studies. Although it is meanwhile known that ADHD often persists even in elderly adults [32], studies on the association between ADHD and suicidal behavior among the general adult population have been rare and inconclusive [44]. While several investigations of the association between ADHD and suicide suggested that the relation was at best modest, and primarily due to ADHD exacerbating the effects of other comorbid conditions [1, 23], recent studies found that there was a strong relation between ADHD and suicide attempts or deaths independent of psychiatric comorbidity [29, 44].

Suicide is among the leading causes of death worldwide but the need for diagnosis and treatment of suicidal behavior is still unmet [19, 36]. Completed suicide, especially in young people is one of the most tragic events to be encountered by a person’s environment. In such cases the question of whether or how such a deed could have been prevented always arises but very often remains unanswered. In young adults, suicidal ideation are obviously a very common event occurring in up to 44 % of high school students [9, 28, 39]. Although suicide is still most frequent in elderly people, an increase in suicide rates among younger people has been observed [51]. A study investigating suicidal behavior of high school students in Vienna, Austria, found suicidal ideation at some point in their lives in as many as 37.9 % of all subjects investigated. A value matching data of other studies performed on suicidality in youth [9, 28, 39]. An investigation of completed suicide in Austrian children and adolescents between 1946 and 2002 showed a mean suicide rate of 6.2 per 100,000 [11] with as many as 1.4 per 100,000 suicides in the group of 10 to 14-year-olds [10].

Very little is known about the interaction of ADHD symptoms and suicidal behavior in young adults. This group however has to be considered at special risk, as only about 15 % of young people with attention deficit hyperactivity disorder (ADHD) make a transition into an adult psychiatric treatment setting [37]. Also, adult psychiatrists have only recently directed their attention to the field of ADHD and adult ADHD services are sparse or non-existent. Many professionals are skeptical about the existence of ADHD in adulthood, and adult psychiatrists most likely do not screen for ADHD in their patients as child and youth psychiatrists do in minors [46]. Another reason that puts young adults with ADHD at special risk is the fact that young adults are, due to neurobiological maturation processes, certainly more prone to act impulsively and are thus more likely to execute suicidal tendencies than older subjects with a comparable psychiatric condition [17].

This investigation set out to examine the association of ADHD with suicidal ideation in a non-clinical sample of 18-year-old males. Additionally, we examined the added effect of comorbid substance use disorder on rates of suicidal ideation in individuals with ADHD.

## Method

### Overview, sample, procedures

In Austria military service is mandatory for all 18-year-old males. Conscription of military servicemen is based on a preliminary psychological and medical examination of all males who turn 18 in the respectable year. This investigation includes blood and urine testing as well as a psychological examination in order to assess the subject’s fitness and capability to perform National Service. In order to obtain an unbiased and representative sample we chose 11 districts including urban and rural areas, agricultural and industrial regions, as well as lower and higher income areas.

### Data collection

Data collection has been performed from January 2010 to December 2010 at two of a total of six recruitment centers for military service in Austria. One of these centers was located in the province of Tyrol, the other one in the province of Lower Austria. All male residents of the selected districts born in 1992, who were liable to enlistment to the Military Service were included in the study. Participation in the study was declined by 60 out of 3340 persons (22 (1.70 %) of 1297 young men from the districts in Tyrol and 38 (1.86 %) of 2043 subjects in Lower Austria). With a response rate of 98.20 %, a total 3280 subjects have thus been enrolled in the investigation. Data were collected in addition to the standard procedure performed by Military Service authorities. Our examination was performed anonymously after the examination for the Military Service, subjects were aware that no information they gave would be communicated to the examiner of the Armed Forces.

### Measures

#### ADHD

Life time prevalence of ADHD was examined using the Wender Utah Rating Scale (WURS-K) [41, 50], an instrument designed for the retrospective diagnosis of attention deficit/hyperactivity disorder in adults. Current symptoms of ADHD were investigated by means of the ADHD checklist for adults based on DSM IV [2].

#### Alcohol use

The frequency of alcohol consumption was reported according to the question: “Do you drink alcohol?” (0) I do not drink, (1) less than once a week, (2) once a week, (3) several times a week, (4) every day. In addition, the CAGE questionnaire (Cutting down, Annoyance by criticism, Guilty feeling and Eye-openers) was used to rate alcohol related behavior. The CAGE questionnaire [13] has good sensitivity and specificity for alcohol dependence [5]. Both alcohol consumption measures were correlated with r = 0.41, *p* < 0.001.

#### Nicotine use

Smoking was assessed with the question: “Do you smoke?” (“Yes” or “No”) followed by the Heavy Smoking Index (HSI), which consists of two questions: “How many cigarettes do you smoke per day?” (“Non-smoker”, “10 or less”, “11–20”, “21–30” and “31 or more”) and “When do you smoke your first cigarette in the morning?” (“Within 5 minutes” “6–30 minutes” “31–60 minutes” and “After more than 60 minutes”). Both questions were scored between 0 and 3 and an HSI-sum score ranging between 0 and 6 was built. The HSI has been previously validated by plasma and saliva cotinine, as well as carbon monoxide levels [20, 21, 24].

#### Illicit drug use

To assess experiences with illicit drugs, the subjects were asked, “Have you ever consumed one of the following drugs?” The response options for each substance (THC, Benzodiazepines, Cocaine, Opiates, Ecstasy, and other drugs) were: (0) never, (1) once, (2) several times (3) regularly. Based on these reports, a dummy variable “drug experience” was build, representing at least one drug experience during lifetime.

#### Suicidal ideation

Suicidal ideation was assessed by the question “Have you ever considered to commit suicide?” with the response options: (0) never, (1) at some earlier point in life, (2) last month, (3) last week. A positive answer (being any answer from 1 to 3) was considered as lifetime suicidal ideation in further analyses.

#### Other variables

To verify reports, GGT, MCV, ALT, AST, were determined in serum samples as were illicit drugs in urine samples Carbon monoxide (CO) levels in exhaled air were determined using a “smokerlyser” (EC50 Smokerlyser; Bedfont Instruments; Kent, UK).

## Statistics

Differences in characteristics between subjects with and without ADHD symptoms were assessed by T‑tests and Mann–Whitney-U tests for not normally distributed variables.

Regression analyses (adjusted for abuse of nicotine, alcohol illicit substances and former ADHD) were used to investigate whether suicidal ideations were associated with prevalence of ADHD. *P*-values at the level of *p* < 0.05 were considered significant. Log-linear analysis was performed to determine a possible synergistic effect of ADHD and drug abuse on suicidal ideations. Data Analysis was conducted using IBM SPSS Statistics 21.0 software (SPSS Inc., Chicago, IL).

## Results

The sample included 3340 18-year-old males of which 3280 consented to participate in the investigation. Out of the total sample 2.7 % stated that they have been treated for ADHD and 1.5 % reported that they had at one point received pharmacological treatment for the condition. Concerning WURS, 10.1 % had a score above the cut-off of >36 indicating past ADHD symptoms. There was a strong association of former (WURS) and current (ADHD checklist) symptoms (r = 0,648, *p* = < 0.001). Alcohol consumption at any rate was very common (91.4 %) with 19.3 % meeting the CAGE cut-off score of 2 or higher, indicating an alcohol problem. Regular smoking was reported by 43.9 %, life time use of cannabis was stated by 19 %. The prevalence of Cocaine abuse was 2.6 % that of benzodiazepine abuse was 2.1 %. Unspecific psychoactive substances have at least once been used by 1.8 % and opiates by 0.2 % of all participants. For details also see Table 1.

**Table 1:** Sample characteristics

	*n*
*Educational status*	
High-school diploma	118
Attending high-school	1389
Basic education (9grades)	1555
Failed to complete 9th grade	46
Special needs school	32
*Financial status of family*	
Very Good	1062
Good	1436
Sufficient	680
*Smoking*	
Non-smoker	1818
Smoker	1421
*HSI Score*	
0–3	1156
4–6	236
*CAGE*	
0	1318
1	1083
≥2	576
*Life time use of illicit Substances*	
Cannabis	618
Benzodiazepines	66
Cocaine	83
Opiates	22
Ecstasy	63
Unspecified psychoactive substances	58

Suicidal ideation at any time was found in 6.8 % of all subjects. Former ADHD symptoms (*p *< 0.001) and drug abuse (*p *< 0.001) significantly predicted suicidal ideations in the subjects investigated. Odds for suicidal behavior increased with the number of symptoms. The log-linear analysis determining the influence of both ADHD an drug abuse on suicidality produced a model with significant two-way-interactions between ADHD and suicidal ideas (χ² (1) = 60.54, *p* < 0.01), between ADHD and drug abuse (χ² (1) = 4.67, *p* = 0.03) and between drug abuse and suicidal ideas (χ² (1) = 20.37, *p* < 0.01), but no significant three-way-interaction (χ² (1) = 0.02, *p* = 0.89). Alcohol and Nicotine abuse did not have any significant influence on suicidality both in subjects with and without ADHD symptoms (Table 2).

**Table 2:** Influence of different factors on suicidal ideations during 12 months prior to examination

		Prevalence of suicidal ideation 95 % CI for Odds Ratio	
	B (SE)	Lower CL	OR
Constant	−4.84	–	–
Former ADHD	0.06 (0.01)***	1.03	1.06
Current ADHD	0.04 (0.03)	0.99	1.04
Abuse of Nicotine	0.47 (0.31)	0.86	1.60
Abuse of Alkohol	0.35 (0.29)	0.80	1.42
Abuse of other drugs	1.16 (0.29)***	1.81	3.20

*p < 0.05; **p < 0.01; ***p < 0.001, R^2^ = 0.11 (Cox & Snell), 0.27 (Nagelkerke), Model χ^2^(5) = 113.61, *p* < 0.01

## Discussion

This study examined the association between ADHD symptoms and suicide ideation in 18-year-old males during their routing investigation for military service. In this sample we found a proportion of 6.8 % reporting lifetime suicidal ideations.

An earlier investigation performed in high school students in the same country [9] and other studies performed in youth in Europe found rates of suicidality between 31 and 44 % [8, 18, 48]. All investigations mentioned above were using self-administered interviews completed anonymously so these differences cannot be explained by different levels of openness. Subjects investigated had also been informed that military authorities would not gain insight in any of the results of the questionnaires completed in the framework of our investigation, so preoccupation about the outcome of their investigation by the armed forces is not likely to impair openness either. A possible explanation however is that subjects in our sample were older than in the above mentioned studies and probably approaching lower values, which are usually found in adults [26]. This idea is supported by the fact that an investigation in a comparable group of non-clinical young adults found a rate of lifetime suicidal ideations of 12.5 % [43]. This generally observable decline of lifetime suicidal ideation with age can probably be explained by a recall bias or a neglect of suicidal thoughts that might have occurred earlier in life.

Clinically relevant former ADHD symptoms were found in 10 %, which can be considered comparable to the percentage estimated for the general population [15]. About one third of the subjects that seemingly fulfilled ADHD criteria has actually been diagnosed with ADHD and received treatment for the condition and roughly half of those who had undergone treatment had been prescribed medication. Lifetime use of alcohol (91.4 %) as well as problem drinking (19.3 %) was consistent with the prevalence in the general population as was the use of illicit substances. Regular smoking was, however, more frequent than expected (43.9 %). Logistic regression analysis showed that there was a positive and statistically significant relation between lifetime suicide ideation and former ADHD symptoms as well as between suicide ideation and abuse of illicit drugs.

Concerning substance abuse, we were surprised not to find an association of smoking or alcohol consumption and suicidal ideation. Abuse of these substances has been identified as independent risk factors for the development of suicidal behavior [9, 52]. While the strong association of drug abuse with suicidal thoughts found in our sample is consistent with other findings [40], we do not have an explanation for the missing influence of nicotine and alcohol on suicidal tendencies.

It has recently been suggested that ADHD is associated with a small but definite increase in the risk of suicide [1, 35]. Mortality rates significantly depended on age at first ADHD diagnosis. People diagnosed with ADHD in adulthood had a greater risk of premature death than those diagnosed in childhood and adolescence [6]. This finding could be caused by the fact that persistent ADHD represents a more severe form of the disorder. An elevated risk for suicidal tendencies during early adulthood could also be explained by a reduction or weaning of stimulant medication, which is also common during adolescence [34]. As adolescence and early adulthood can be considered times of great risk for the development of suicidality especially during psychosocial stress [26], this obviously concerns individuals affected by ADHD to an even greater extent. Some studies, however, that were examining the association between ADHD and suicidal behavior among the general adult population have found an association with suicidal tendencies that was only weak to moderate and also considered comorbid mental disorders to play an important role in the development of suicidal behavior [1]. While methodological differences between investigations on suicidality and ADHD render comparability of many studies difficult, two recent investigations examining the connection of ADHD and suicidal ideations in large samples of the general population both found that adults with more ADHD symptoms had significantly higher odds for suicidal behavior, the risk remaining substantial even after adjusting for comorbid disorders [29, 44]. In our sample, former, but not current ADHD syndrome correlated with an increased risk of suicidal thoughts. This might be explained by the fact that subjects scoring positive for childhood ADHD might suffer from a more pronounced form of the disease. It is also important to bear in mind that of all subjects scoring positive for childhood ADHD, only less than one third has been diagnosed with the disease. Therefore, fulfilling the diagnostic criteria in this sample is not equivalent with ever having been diagnosed with ADHD.

In consideration of possible reasons for the connection of ADHD and suicidal thoughts in young adults, the trait of impulsiveness [16] might be crucial. Impulsiveness is the tendency to act quickly without prior thought and with little regard for the consequences. It is one of the central components in ADHD [3, 33] but has also been described to be associated with an increased risk for suicidal behavior [30]. Cyclothymic temperament as another possible link is highly prevalent in adults with ADHD [27] and has also been shown to correlate with suicidal tendencies [43]. Self-directedness, reward dependence, persistence and cooperativeness as features commonly associated with a resilient profile negatively correlate with ADHD [31].

Currently, a trend towards the revision of nosologic criteria and deconstruction of global assessments of ADHD into component subtypes can be observed [25] by elaborating subgroups and focusing on specific traits, identification of subjects at risk for suicidal tendencies amongst those affected by ADHD might be facilitated.

## Conclusion

Although suicide prevention programs and research on factors contributing to suicidal behavior have been extensive for many decades, current research on suicide prevention in subjects suffering from ADHD is limited. The finding that young adults in the general population with a history of ADHD have an increased risk for suicide ideation has several implications. On the one hand prospective studies on the interaction of ADHD and suicidal behavior in order to better understand the relationship between ADHD and suicidal behavior in this age group are needed. On the other hand, preventive strategies focusing on young adults with a history of ADHD ought to be established.

## Limitations and strengths

As always, the results of this study must be interpreted in the context of several limitations. Firstly, the sample consists of men only. Secondly, as subjects had to be fit enough to perform military service, men with severe mental or physical disorders were excluded, thus generating a possible bias towards the inclusion of subjects healthier than in the general population. Finally, the cross-sectional study design does not allow making conclusions about the temporal relationships between ADHD symptoms and suicidality.

In terms of strengths our sample is highly representative including 6.8 % of all males born in Austria in 1992 all 18-year-old males of the respective areas fit enough to be enlisted for military service. Districts of investigation were carefully chosen in order to obtain an unbiased and representative sample. All subjects were rated by one single person, who was very experienced in all questionnaires applied. We have employed widely used questionnaire instruments with recognized reliability and validity. Reports on substance use were verified by examination of blood, urine and breath.
